# miR-422a inhibits cell proliferation in colorectal cancer by targeting AKT1 and MAPK1

**DOI:** 10.1186/s12935-017-0461-3

**Published:** 2017-10-28

**Authors:** Wen-Ting Wei, Xin-Xin Nian, Shu-Yang Wang, Hong-Li Jiao, Yong-Xia Wang, Zhi-Yuan Xiao, Run-Wei Yang, Yan-Qing Ding, Ya-Ping Ye, Wen-Ting Liao

**Affiliations:** 1 Department of Pathology, Nanfang Hospital, Southern Medical University, Guangzhou, 510515 Guangdong China; 2 Department of Pathology, School of Basic Medical Sciences, Southern Medical University, Guangzhou, Guangdong China; 3 Guangdong Provincial Key Laboratory of Molecular Tumor Pathology, Guangzhou, Guangdong China

**Keywords:** miR-422a, Proliferation, Prognosis, Colorectal cancer, AKT1, MAPK1

## Abstract

**Background:**

miRNAs are regarded as molecular biomarkers and therapeutic targets for colorectal cancer (CRC), a series of miRNAs have been proven to involve into CRC carcinogenesis, invasion and metastasis. Aberrant miR-422a expression and its roles have been reported in some cancers. However, the function and underlying mechanism of miR-422a in the progression of CRC remain largely unknown.

**Methods:**

Real-time PCR were used to quantify miR-422a expression in CRC tissues. Both vivo and vitro functional assays showed miR-422a inhibits CRC cell proliferation. Target prediction program (miRBase) and luciferase reporter assays were conducted to confirm the target genes AKT1 and MAPK1 of miR-422a. Specimens from 50 patients with CRC were analyzed for the correlation between the expression of miR-422a and the expression of the target genes AKT1 and MAPK1 by real-time PCR.

**Results:**

MiR-422a was down‑regulated in CRC tissues and cell lines. Ectopic expression of miR-422a inhibited cell proliferation and tumor growth ability; inhibition of endogenous miR-422a, by contrast, promoted cell proliferation and tumor growth ability of CRC cells. MiR-422a directly targets 3′-UTR of the AKT1 and MAPK1, down-regulation of miR-422a led to the activation of Raf/MEK/ERK and PI3K/AKT signaling pathways to promote cell proliferation in CRC. In addition, miR-422a expression was negatively correlated with the expressions of AKT1 and MAPK1 in CRC tissues.

**Conclusion:**

miR-422a inhibits cell proliferation in colorectal cancer by targeting AKT1 and MAPK1.

**Electronic supplementary material:**

The online version of this article (doi:10.1186/s12935-017-0461-3) contains supplementary material, which is available to authorized users.

## Background

Colorectal cancer (CRC) is one of the most common cancer types which shows high morbidity and mortality [1]. The past decades have seen decreasing mortality of CRC as the early detection and treatment have advanced greatly, but the incidence of CRC increases worldwide and the onset age is becoming younger [1–3]. Hence, it is still imminent to further clarify the exact pathogenesis of CRC. Multiple studies have been conducted to investigate the mutation of genes and their products [4–7], which prove that the aberrant activation of signaling pathways [8–11] and microsatellite instability (MSI) [7, 11] are involved in oncogenesis and progression of CRC. Moreover, recent studies indicate that the regulation of micro-RNAs (miRNAs) is indispensable [12–14].

Raf/MEK/ERK and PI3K/Akt are both signal transduction pathway that regulate intracellular processes in response to extracellular signals. ERK and AKT are, respectively, the key proteins of Raf/MEK/ERK pathway and PI3K/Akt pathway. The aberrant activation of Raf/MEK/ERK and PI3K/Akt signaling pathway is considered to be an essential issue in tumorigenesis and progression of CRC.

miRNAs are a class of small-regulatory RNA molecules, which are highly conserved across species. MiRNAs regulate gene expression through binding to the 3′-untranslated region (UTR) of their target mRNAs in a sequence-specific manner [15]. In recent years, miRNAs are regarded as molecular biomarkers and therapeutic targets for CRC. A series of miRNAs have been proven to involve into CRC carcinogenesis, invasion and metastasis [12, 16]. For example, MicroRNA-30b can function as a tumor suppressor in CRC by targeting KRAS, PIK3CD and BCL2 [17], while MicroRNA-224, a tumor promoter, targets PHLPP1 and PHLPP2 [18], sustains Wnt/β-catenin signaling and promotes aggressive phenotype of CRC [19]. Besides, many other micro-RNAs such as miR-30a [20], miR-140-5p [21] and miR-153 [22] are also known as important moderators in the progression of CRC. However, a large number of functional miRNAs remains to be investigated in CRC [23].

Several studies have indicated the miR-422a takes part in many human diseases such as postmenopausal osteoporosis, osarcoma and colorectal adenocarcinoma [24–26]. Moreover, miR-422a plays a positive role on head and neck squamous cell carcinoma by targeting NT5E/CD73 that promotes loco-regional recurrence, miR-422a were also found to significantly inhibit TMEM45B expression in squamous cell lung cancer [27, 28]. Recent studies illuminate that miR-422a is associated with advanced stages of CRC, affects G1/S transition and potentially inhibits hTERT expression in CRC, which suggests miR-422a to be an independent prognostic factor of CRC [29–31]. However, the more other target genes and underlying mechanism of miR-422a in the progression of CRC are largely unknown.

In this study, we report that miR-422a is down‑regulated in CRC tissues and cell lines; ectopic expression of miR-422a inhibits cell proliferation and tumor growth ability, inhibition of endogenous miR-422a, by contrast, promotes cell proliferation and tumor growth ability of CRC cells; miR-422a directly targets 3′-UTR of the AKT1 and MAPK1, down-regulation of miR-422a led to the activation of Raf/MEK/ERK and PI3K/AKT signaling pathways to promote cell proliferation in CRC.

## Methods

### Tissue specimens and cell cultures

The 30 freshly CRC specimens and their matched adjacent normal tissues frozen and stored in liquid nitrogen until further use were collected from operation room of Nanfang Hospital. Prior approval was obtained from the Southern Medical University Institutional Board (Guangzhou, China) before using these clinical materials for research. All samples were collected and analyzed with the prior written, informed consent of the patients. Four human CRC lines SW620, SW837, HCT15 and HCT116 were purchased from American Type Culture Collection Cell Biology Collection and were cultured in RPMI-1640 medium (Gibco, Grand Island, NY, USA) containing 10% fetal bovine serum (FBS; PAA Laboratories, Pasching, Austria) at 37 °C with 5% CO_2_.

### Plasmids and transfection

The miR-422a mimic, miR-422a inhibitor and the negative control were obtained from RiboBio (Guangzhou, China) and transfected into CRC cells using Lipofectamine 2000 reagent (Invitrogen). The full length of MAPK1 3′-UTR contains 4593 bps and the AKT1 3′-UTR has 1011 bps. The miR-422a binding site in the MAPK1 3′-UTR is located at 176–188 bps, while 411–425 bps in the AKT1 3′-UTR. The region of the human MAPK1 3′-UTR from 172 to 191 bps and AKT1 3′-UTR from 407 to 429 bp were generated by PCR amplification and subcloned into the MluI/NheI sites of the pGL3-basic luciferase reporter plasmid (Promega). The primers are listed in Additional file 1: Table S1.

### RNA isolation, reverse transcription (RT) and real-time PCR

Total RNA from cell lines, CRC tissues and normal mucosa was extracted using Trizol reagent (Invitrogen). The cDNA was reverse-transcribed at 37 °C for an hour and 85 °C for 5 min in a 10 µl reaction system including 3.75 μl (0.25–8 μg) RNA sample, 5 μl mRQ Buffer (2×), 1.25 μl mRQ Enzyme (takara, Guangzhou, China), then add 90 μl H_2_O to bring the total volume to 100 μl. These cDNAs were ready for the miRNA quantification according to the protocol (takara Guangzhou, China). The PCR reaction was performed as follows: 95 °C for 10 s, 40 cycles of 95 °C for 5 s and 60 °C for 20 s and 95 °C for 60 s, 55 °C for 30 s, and 95 °C for 30 s. Sequences of the primers are summarized in Additional file 2: Table S2.

### Western blotting

We carried out western blotting according to standard methods, which protein lysates were prepared, subjected to SDS–PAGE and transferred onto PVDF membranes, using anti-AKT (Cell Signaling Technology, Danvers, MA, USA), anti-ERK1/2 (CST, USA), anti-GSK-3β (CST, USA), anti-p21, anti-p27, anti-CyclinD1 (Bioworld Technology, St. Louis Park, MN, USA), anti-p-AKT (CST, USA), anti-p-GSK-3βand anti-p-ERK1/2 (CST, USA). Anti-α-Tubulin (Sigma, St. Louis, MO, USA) monoclonal antibody served as a internal reference.

### MTT assay, colony formation assay, soft-agar colony formation assay, flow cytometry and luciferase assays

The miR-422a mimics, anti- miR-422a inhibitors and negative control oligos were transiently transfected into CRC cells for the MTT assay, colony-formation assay, soft agar colony-formation assay, flow-cytometry and luciferase assays, as previously described [32]. Further details are described in Additional file 3: Additional materials and methods. All experiments were repeated at least three times and the data are presented as mean ± SD.

### Tumorigenesis assay

200 μl cell suspension (2 × 10^6^ cells) prepared with 0.9% normal saline were subcutaneously injected into 4-week-old Balb/Cathymic nude mice (nu/nu) bought from the Animal Center of Southern Medical University, Guangzhou, China. Tumor volumes were measured on the indicated days. Details refer to studies described previously [18, 33].

### Statistical analysis

All statistical analyses were analyzed using SPSS21.0 for Windows. The independent-samples t test and paired-samples t-test were used to analyze two groups. The correlation analysis was used to explain the relationship between miR-422a expression and its target gene. *p* < 0.05 was considered statistically significant.

## Results

### MiR‑422a is down‑regulated in CRC tissues

**Fig. 1:**
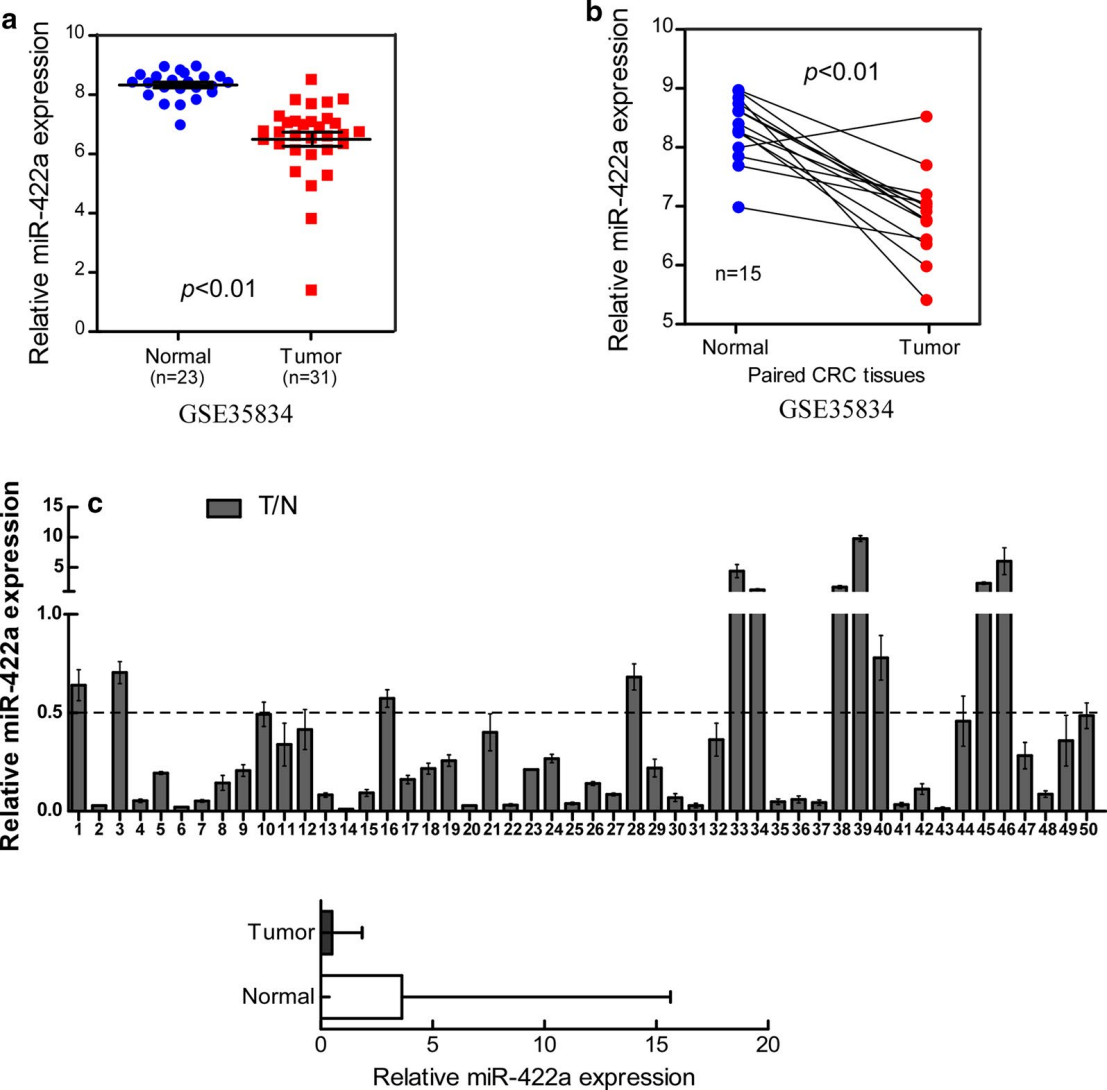
MiR‑422a is down‑regulated in CRC tissues (*p* < 0.01). MiR-422a expression was assessed by real-time PCR and was normalized by U6 expression. The bounds of boxes represent the lower and upper quartiles. Lines within boxes and whiskers denote median and extremum, respectively. **a** The expression of miR-422a in the 31 human CRC tissues and 23 normal intestine epithelial tissues from GEO database (GSE35834). **b** Mean expression of miR-422a in 15 paired tissues (T and N) from GEO database (GSE35834). **c** The relative expression of miR-422a in the 50 human CRC tissues (T) and their paired normal colorectal tissues (N) acquired from Nanfang Hospital

We ascertained the expression of miR-422a in published CRC miRNA microarray datasets (GEO, GSE35834) (Additional file 4: Tables S3 and S4). We found that miR-422a was down-regulated in CRC (Fig. 1a, *p* < 0.01), and 93.3% (14/15) tumor tissues had the lower expression of miR-422a than their paired non-cancerous colorectal tissues (Fig. 1b). To further confirmed the results, we tested the expression of miR-422a by real-time PCR analysis in **50** CRC biopsies obtained from Nanfang Hospital. The real-time PCR result was consistent with the GEO database analysis. miR-422a expression was down-regulated in **78% (39/50)** of the CRC tissue samples examined (N/T > two-fold) compared to their matched adjacent normal tissues (p < 0.001, Fig. 1c). Taken together, these results indicate that miR-422a is down‑regulated in CRC tissues.

### Ectopic expression of miR-422a inhibits cell proliferation and tumor growth ability of CRC cells

**Fig. 2:**
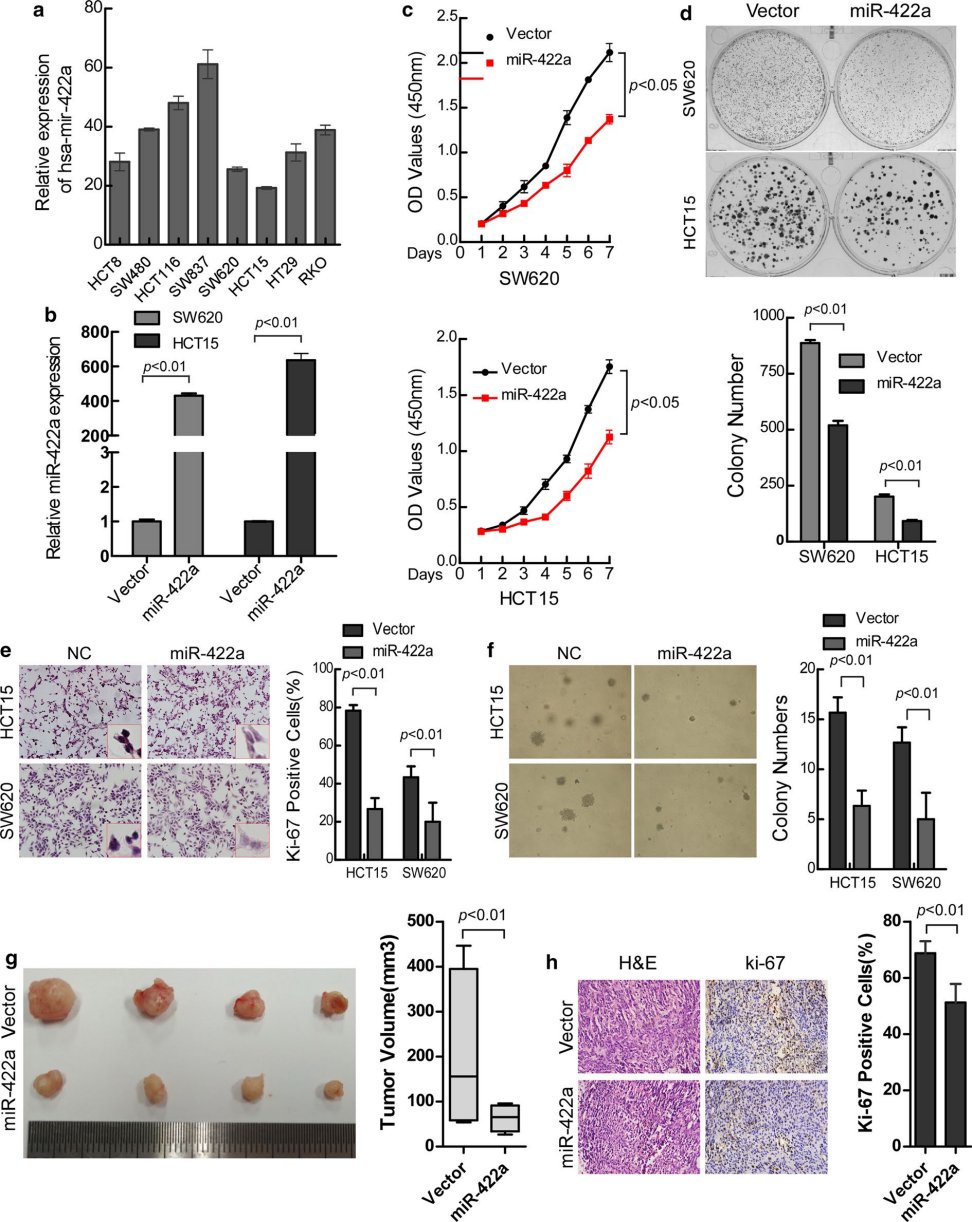
Over-expression of miR-422a inhibits proliferation and tumor growth of CRC cells. **a** miR-422a expression assessed by real-time PCR in eight CRC cell lines. **b** Ectopic expression of miR-422a in SW620 and HCT15 CRC cells was validated by real-time PCR. **c** Cell growth analyzed by MTT assays. **d** Representative results of colony formation. The numbers of colonies containing > 50 cells were scored. The number of colonies counted was of an entire well and the error bars represent mean ± SD from three independent experiments. **e** Quantification of the numbers of Ki-67-positive cells (yellow–brown) by immunohistochemical staining. **f** Anchorage-independent colony-formation assays. Only cell colonies > 0.1 mm in diameter were counted. **g** SW620-Vector and SW620-miR-422a cells were injected into the hind limbs of nude mice (n = 4). Tumor volumes were measured on the indicated days. Data points of tumor volume are presented as the mean ± SD. **h** Histopathological analyses of xenograft tumors. The tumor sections were stained with H & E or subjected to IHC staining using an antibody against Ki-67. Error bars represent mean ± SD from three independent experiments

We observed differential expression of miR-422a in 8 CRC cell lines, which showed that HCT15 and SW620 had a relative lower expression, while SW837 and HCT116 had relative a higher level of miR-422a (Fig. 2a). To explore candidate effects of miR-422a in the progression of CRC, miR-422a was over-expressed in HCT15 and SW620 cells by transfected mimics (Fig. 2b). MTT and colony-formation assay showed that over-expression of miR-422a significantly decreased the growth rate of SW620 and HCT15 cells compared with negative control (NC)-transfected cells (Fig. 2c, d; p < 0.01). Additionally, the proliferation index (calculated by Ki-67 expression) was obviously reduced in miR-422a over-expressing compared with negative control (NC) transfected cells (Fig. 2e).

Then soft agar assay was used to examine the effect of miR-422a on the anchorage-independent growth ability of CRC cells. The result showed that over-expression of miR-422a dramatically decreased the growth of SW620 and HCT15 cells (Fig. 2f; p < 0.01). To further confirm this effect in vivo, we established stable miR-422a over-expressing SW620 cells and performed a tumorigenesis assay in nude mice. As shown in Fig. 2g, the tumor in the treated group (injected with miR-422a over-expressing SW620 cells) grew more slowly than those in the SW620-Vector group. Moreover, lower Ki-67 percent was observed in miR-422a over-expressing group than tumors from the control group by immunostaining (Fig. 2h).

### Inhibition of endogenous miR-422a promotes cell proliferation and tumor growth ability of CRC cells

**Fig. 3:**
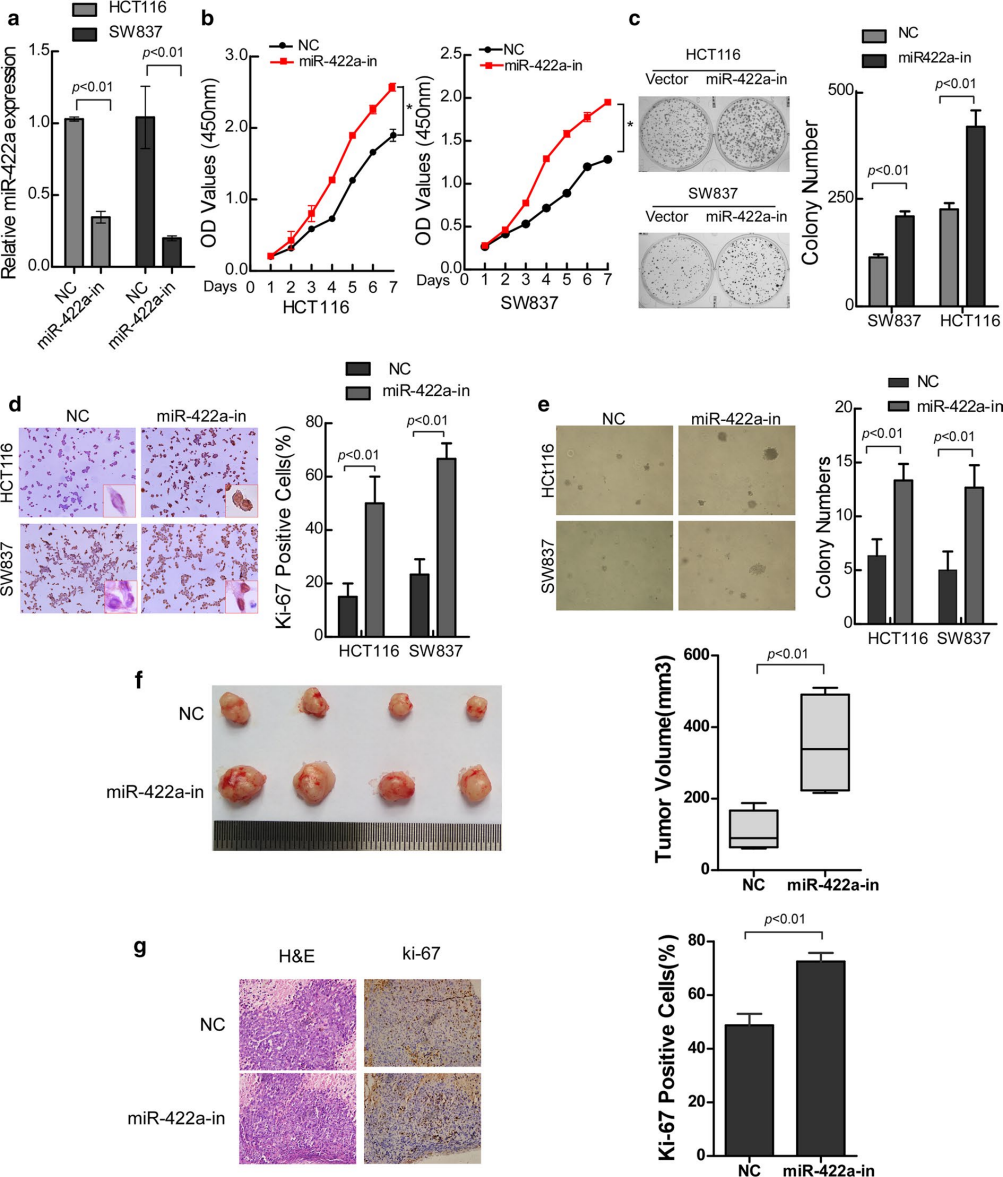
Inhibition of miR-422a promotes proliferation and tumor growth of CRC cells. **a** Ectopic expression of miR-422a-inhibitor in SW837 and HCT116 CRC cells was validated by real-time PCR. **b** Cell growth analyzed by MTT assays. **c** Representative results of colony formation. The numbers of colonies containing > 50 cells were scored and the error bars represent mean ± SD from three independent experiments. **d** Quantification of the numbers of Ki-67-positive cells (yellow–brown) by immunohistochemical staining. **e** Anchorage-independent colony formation assays. Only cell colonies > 0.1 mm in diameter were counted. **f** Images of tumor from nude mice injected with HCT116. (n = 4). Tumor volumes were measured on the indicated days. Data points of tumor volume are presented as the mean ± SD. **g** Histopathological analysis of xenograft tumors. The tumor sections were stained with H & E or subjected to IHC staining using an antibody against Ki-67. Error bars represent mean ± SD from three independent experiments. *p < 0.05

We next suppressed miR-422a in SW837 and HCT116 by transfected miR-422a inhibitors (Fig. 3a). Increased growth rate was significantly displayed in miR-422a-suppressing group compared with the negative control (NC) group in SW837 and HCT116 cells by MTT and colony-formation assay (Fig. 3b, c). Similarly, the proliferation index was significantly increased in miR-422a-suppressing cells compared with the control group (Fig. 3d). Moreover, suppression of endogenous miR-422a resulted in a significant increase in colony number and size in soft agar (Fig. 3e; p < 0.01). Additionally, in order to observe the inhibition effects of miR-422a on tumor growth, we established stable miR-422a-inhibitor SW837 cells (Fig. 3f) and injected them into the hind limbs of nude mice subcutaneously. Consistent with above results, the tumor in the SW837-miR-422a-inhibitor group grew much faster than those in the SW837-NC group. Furthermore, Immunohistochemistry (IHC) confirmed that tumours of the miR-422a-inhibitor displayed much higher Ki-67 percent than tumors of the control group (Fig. 3g).

### MiR-422a prohibits cell cycle progression in CRC cells

**Fig. 4:**
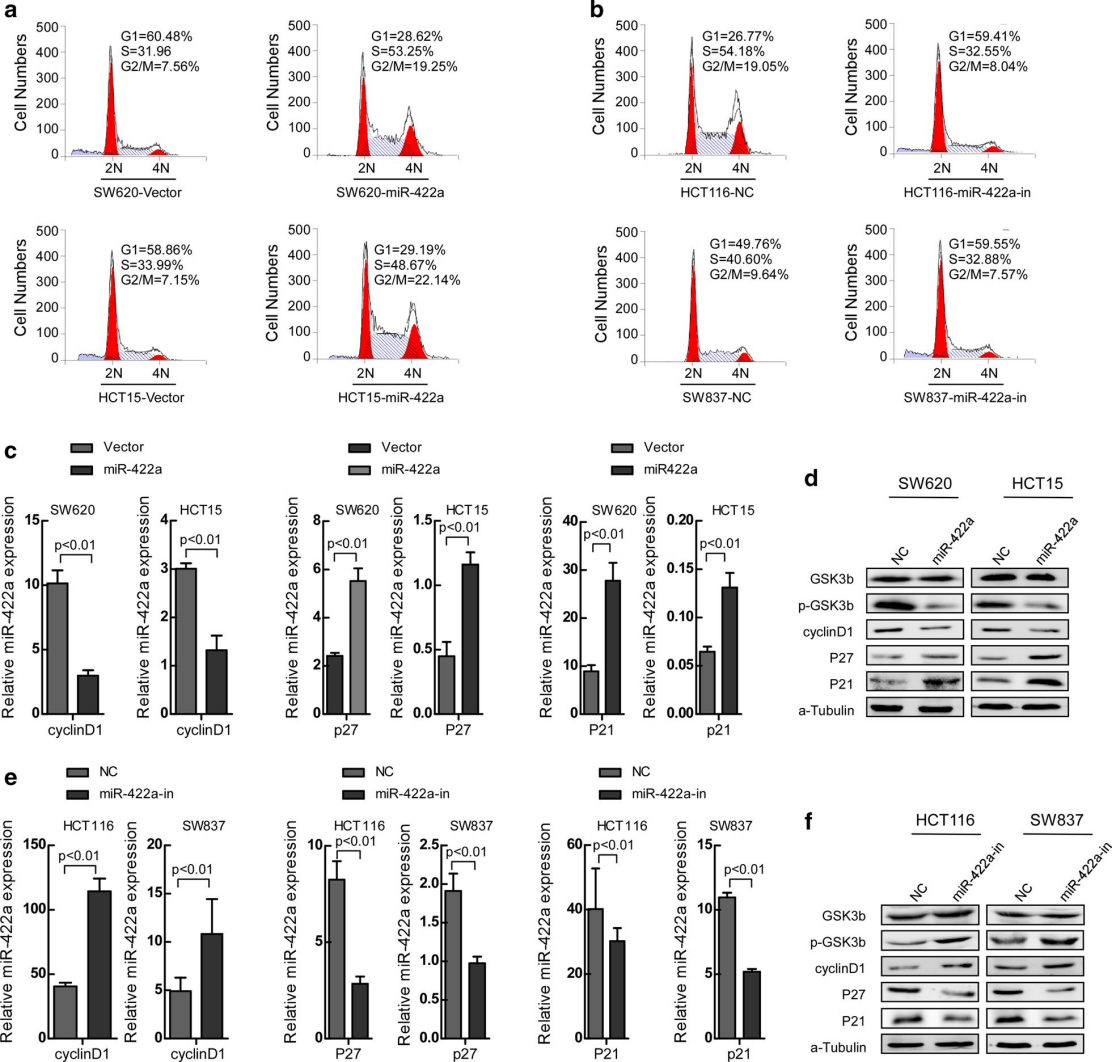
MiR-422a prohibits cell cycle progression by inhibiting the activity of Raf/MEK/ERK and PI3K/AKT signaling pathways. **a**, **b** Flow-cytometry analyses of the cell cycle of the indicated CRC cells synchronized in the G2/M phase by treatment with 0.1 µM colchicine for 12 h. Error bars represent mean ± SD from three independent experiments. **c**, **e** Real-time PCR analysis of CyclinD1, p21 and p27 in the indicated cells. **d**, **f** Western blot analysis of GSK-3β, p-GSK-3β, CyclinD1, p21 and p27 in the indicated cells

To explore the possible mechanism by which miR-422a regulates the proliferation and tumor growth of human CRC cells, we determined the distribution of cells within the stages of the cell cycle by flow cytometry. Cells treated with miR-422a mimics induced a significant increase in the percentage of cells in the G1/G0 peak and a decrease in the percentage of cells in the S and G2/M peaks (*p* < 0.01) (Fig. 4a, Additional file 5: Figure S1A). However, miR-422a-inhibitor cells led to a dramatic decrease in the percentage of cells in the G1/G0 peak and an increase in the S and G2/M peaks (*p* < 0.01) (Fig. 4b, Additional file 5: Figure S1B). Additionally, we also detected the expression levels of a number of critical cell-cycle regulators. Results showed that phosphorylation levels of GSK3β and cyclin D1 were significantly down-regulated, whereas p21^Cip1^ and p27^Kip1^ were strikingly up-regulated at the protein levels in miR-422a over-expressing cells, and all these results were opposite in miR-422a suppressing cells. But no obvious change were found on total GSK3β (Fig. 4c–f). Thus, we hypothesized that up-regulation of miR-422a may devitalize AKT/GSK3β and ERK/GSK3β signaling.

### MiR-422a directly targets AKT1 and MAPK1 to regulate the activity of the Raf/MEK/ERK and PI3K/AKT signaling pathways

**Fig. 5:**
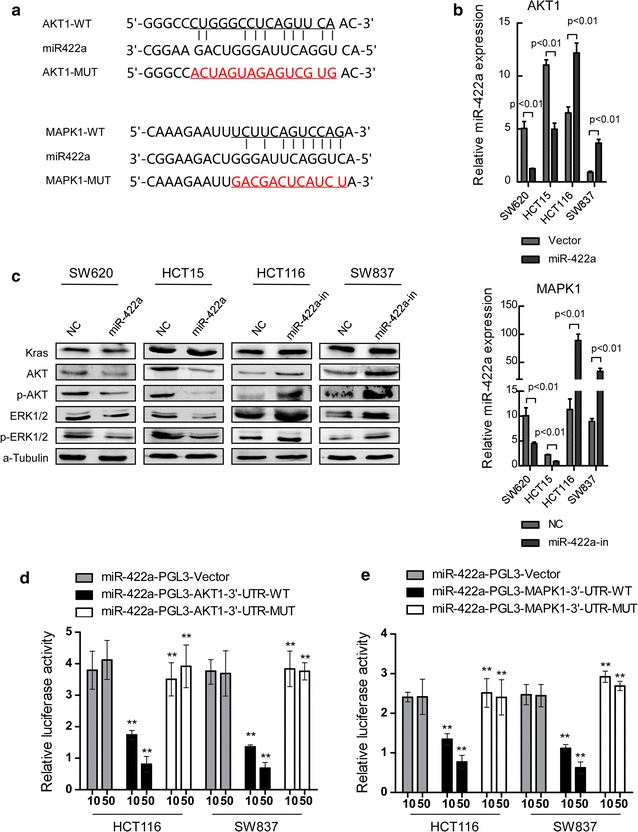
MiR-224 directly targets AKT1 and MAPK1. **a** Predicted miR-422a target sequences in the 3′-UTRs of AKT1 and MAPK1, and their mutants containing altered nucleotides in the 3′-UTRs. **b** Real-time PCR analysis of AKT1 and MAPK1 in the indicated cells. **c** Western blot analysis of AKT1 and MAPK1, and the their phosphorylation in the indicated cells. **d**, **e** Luciferase assay analyses of the indicated cells transfected with the indicated reporters with increasing amounts of miR-422a (10 and 50 nM). Error bars represent mean ± SD from three independent experiments.**p < 0.01

In order to explore the potential mechanism, target prediction program (miRBase) to analyze the target genes of miR-422a. Resut showed that AKT1 and MAPK1 were highly concerned. The 3′-UTR of AKT1 and MAPK1 mRNA contains a complementary sequence for the seed region of miR-422a (Fig. 5a). AKT1 (also known as AKT) and MAPK1 (also known as ERK2) were reporetd to be important **c**ell-cycly factors, which involved in regulation of p27 ^Kip1^, p21^Cip1^, cyclinD1 and GSK3β.

As shown in Fig. 5b and c, over-expression of miR-422a led to decreased phosphorylation levels of both ERK1/2(MAPK1) and AKT in CRC cells. Interestingly, expression level of total ERK1/2 and AKT were also decreased by over-expression of miR-422a. And all these results were opposite in miR-422a suppressing cells. These results point out that down-regulation of miR-422a might regulate the activity of the Raf/MEK/ERK and PI3K/AKT signaling pathways to promote the progression of CRC. Besides, we respectively detected the expression of KRAS, which involved into regulation of AKT/GSK3β and ERK/GSK3β signaling, in miR-422a–overexpressing cells and miR-422a–inhibiting cells. The expression of KRAS was not observed significant change.

Furthermore, we subcloned the wild-type and mutant fragments of AKT1 or MAPK1 3′-UTR fragment separately into the pGL3-basic luciferase reporter vectors. As showed in Fig. 5d and e, both wild-type AKT1 and MAPK1 reporter gene luciferase activity was dose-dependent reduced upon dose-dependently overexpression of miR-422a in both CRC cell lines, whereas inhibition of miR-422a increased wild-type AKT1 or MAPK1 luciferase activity. However, mutations in the tentative miR-422a-binding seed region in AKT1 or MAPK1 3′-UTRs abrogated the suppressive effect on AKT1 or MAPK1 mediated by miR-422a. These results demonstrated that miR-422a could directly target AKT1 and MAPK1 in CRC cells and regulated cell-cycle regulators: p27 ^Kip1^, p21^Cip1^ and cyclinD1.

### miR-422a expression negatively correlated with the expressions of AKT1 and MAPK1 in CRC tissues

**Fig. 6:**
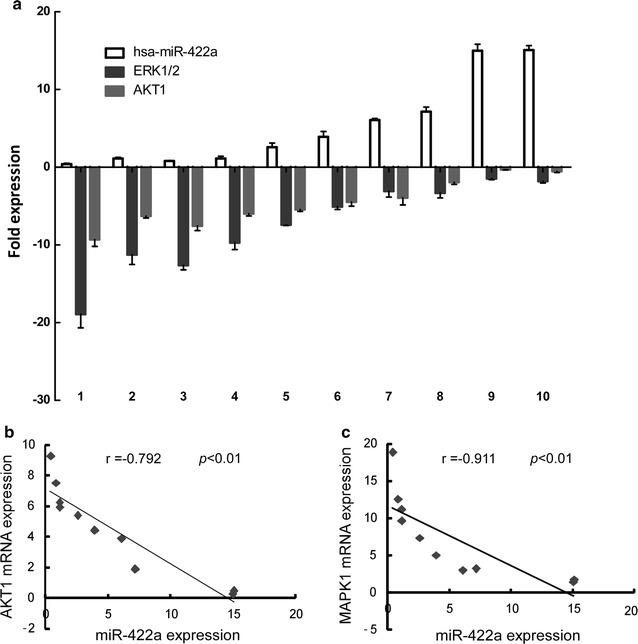
miR-422a expression negatively correlated with the expressions of AKT1 and MAPK1 in CRC tissues. **a** Real-time PCR analysis of miR-422a, AKT1 or MAPK1 expressionand. **b**, **c** Spearman correlation analyses between relative miR-422a expression and relative mRNA expression levels of AKT1 or MAPK1 in ten fresh human CRC samples

To further verify the above findings, we first analyzed ten pairs of fresh CRC tissues and paired normal tissues to explore the relationship between miR-422a and the target genes AKT1 or MAPK1. Figure 6a revealed that miR-422a was down-regulated in CRC tissues while AKT1 or MAPK1 were up-regulated in CRC tissues (Additional file 4: Table S3). Spearman correlation analyses showed that miR-422a expression negatively correlated with the expressions of AKT1 (r = − 0.792, p < 0.01) and MAPK1 (r = − 0.91, p < 0.01) (Fig. 6b and c).

## Discussion

It is known that miRNAs function as suppressive or carcinogenic factors in a variety of cancers [34]. For example, miR-638 is an important tumour suppressor [35], while miR-153 represents an onco-microRNA in CRC [22]. Previous findings suggested that miR-422a might work as a tumor suppressor [36] in glioblastoma and hepatocellular carcinoma involving in a feedback loop with family of forkhead box (FOX) [37]. Recently, miR-422a have been found to function as a protector against CRC because the expression level of miR-422a is lower in CRC tissue compared with normal tissue, and it is revealed that miR-422a may be relevant with cell proliferation [31]. But the mechanism of how miR-422a inhibit cell proliferation and in the progression of CRC remain to be elucidated. Our results indicated that miR-422a was down-regulated in human CRC, induced G1 arrest and inhibited cell proliferation and tumour growth thought regulating the activity of Raf–MEK–ERK and PI3K–AKT signaling pathways.

The disorder of the cell cycle is largely responsible for uncontrolled cell proliferation, which is the basic feature of cancer. There are several different cyclins that are activated in different parts of the cell cycle and that cause the CDKs, the key regulatory proteins transforming cell cycle phases, to phosphorylate different substrates. These cyclins are divided into two main groups: G1/S cyclins including cyclin A, Cyclin D and Cyclin E, and G2/M cyclins. The three D type cyclins (cyclin D1, cyclin D2, cyclin D3) bind to CDK4 and to CDK6 and regulate transition from G1 to S phase [38, 39]. Aberrant cyclin D1 expression has been reported in many human cancers [38]. Cyclin D1 is firstly induced by Ras, a small GTP-binding protein, and CDK-cyclin D1 complexes are essential for entry into S phase from G1 phase [40, 41]. CDK activity can be counteracted by CDK inhibitors (CKI) that has distinct two families. Cip/Kip family belongs to CKI, including p21 (Waf1, Cip1), p27 (Cip2), p57 (Kip2) [42, 43]. Our study found that miR-422a up-regulated expression of p27 and p21 and inhibited expression of cyclin D1, arresting the CRC cells at G1/G0 phase and suppressing CRC proliferation.

Cell cycle is regulated by the ubiquitin pathway [44], p53-p21-DREAM-CDE/CHR pathway [45], WNT signaling pathway [46, 47] and so on. The Raf/mitogen-activated protein kinase (MAPK) kinase (MEK)/extracellular signal-regulated kinases (ERK) pathway [48] and Ras/PI3K/AKT pathways [49] are also involved in cell cycle regulation, when Ras actives both Raf and PI3K which further respectively active ERK and AKT [50]. Activation of AKT and ERK can phosphorylate GSK3β, preventing phosphorylation of cyclin D1 which leads to cell cycle progression [51–54]. Mitogen-activated protein kinase (MAPK) is protein kinases and three MAPK families have been clearly characterized, namely classical MAPK (also known as ERK), C-Jun N-terminal kinse/stress-activated protein kinase (JNK/SAPK) and p38 kinase [55]. Since ERK1(MAPK3) and its close relative ERK2 (MAPK1) are both involved in growth factor signaling, the family was termed “mitogen-activated”. We have confirmed that miR-422a reduced total and phosphorylation level of ERK1/2 and AKT1. Numbers of researches have shown that microRNA participates in regulation of these signal pathways. For instance, miR-224 directly targets GSK3β and SFRP2 and activates the Wnt/β-catenin signaling in CRC cells [19]. And miR-1 was reported as a tumor suppressor that restrained epithelial-mesenchymal transition and metastasis of colorectal carcinoma via the MAPK and PI3K/AKT pathway [56].

Indeed, KRAS gene is mutated in nearly 50% of CRCs [57], which is activated after the extracellular mitogen binds to the membrane receptor and leads to aberrant activations of downstream pro-survival signaling cascades including Raf/MEK/ERK, PI3K/PKB(AKT), and Ral-GTPase active MAPK/GSK pathway [58]. Importantly, we found that expression of KRAS was not significantly changed by miR-422, but a dual inhibition of the Raf/MEK/ERK and PI3K/AKT signaling pathways was mediated by miR-422a, binding to the 3′UTR of MAPK1(ERK2) and AKT1 mRNAs. The anti-proliferative molecule GSK3β is an essential regulator of the intrinsic apoptotic pathway, and has been implicated in the development and progression of CRC [51]. GSK3β is also a downstream effector of ERK2 or AKT1 [50]. The present study reveals that miR-422a increased GSK3β expression through regulation of RAS/Raf/MEK–/RK and RAS/PI3K/AKT and finally inhibited cells proliferation in CRC. It has been documented that the expression of p27 ^Kip1^, p21^Cip1^ and cyclinD1 can be transcriptionally regulated by GSK3β and, in turn, the transcriptional activity of GSK3β is modulated by AKT and ERK phosphorylation [9, 42, 43, 59–61].

## Conclusions

Collectively, our results indicated that miR-422a was down-regulated in human CRC. MiR-422a inhibits cell proliferation in colorectal cancer by targeting AKT1 and MAPK1. Down-regulation of miR-422a could promote CRC growth through enhancing the activity Raf/MEK/ERK and PI3K/AKT signaling pathways. Restoration of miR-422a might be a useful therapeutic approach for CRC treatment. However, the detailed mechanism of miR-422a in CRC progression needs further investigations.

## Additional files

**Additional file 1: Table S1.** Primer sequences used for amplification and plasmid construction (5′ to 3′).

**Additional file 2: Table S2.** Primer Sequences Used for Real-time PCR (5′ to 3′).

**Additional file 3.** Additional methods.

**Additional file 4: Table S3.** Characteristics of 46 patients from dataset (GSE35834). **Table S4.** Sample set description.

**Additional file 5: Figure S1.** Statistical analyses of flow cytometry indicated that overexpression of miR-422a caused the arrest of G1-S phase transition (A), while knockdown of miR-422a promoted the G1-S phase transition (B). Error bars represent mean  ±  SD from 3 independent experiments. * p  <  0.01.

## Abbreviations

CRC: colorectal cancer
GSEA: Gene set enrichment analysis
miR-422a: microRNA 422a
AKT1: AKT serine/threonine kinase 1
MAPK1: Mitogen-activated protein kinase 1
RT: reverse transcription
RT-qPCR: Real time quantitative polymerase chain reaction
IHC: immunohistochemistry
CDK: cyclin-dependent kinase
p21: p21Cip1/WAF1
p27: p27Kip1
