# Supplementary material for: miR-422a inhibits cell proliferation in colorectal cancer by targeting AKT1 and MAPK1
Source: Cancer Cell Int. 2017 Oct 28;17:91. doi: 10.1186/s12935-017-0461-3 (PMC5664829; doi:10.1186/s12935-017-0461-3)
Supplement: Supplementary file 1 — Additional file 1: Table S1. Primer sequences used for amplification and plasmid construction (5′ to 3′). [file 12935_2017_461_MOESM1_ESM.doc]

**Supplementary Table S1:** Primer sequences used for amplification and plasmid construction (5’ to 3’)

| **Gene** | **Forward primer** | **Reverse primer** |
| --- | --- | --- |
| MiR-422a | GGACTTAGGGTCAGAAGGC | mRQ3’ primer from reagent kit(200rxn;Cat.No.639676,Takara) |
| AKT1-3’UTR-WT | CAGTTTGTTCTCCGGGTGTGG | TGGGTAAACCCTGGCCCATC |
| MAPK1-3’UTR-WT | CTGGACGTGCTCAGACATCG | GGTCAGCAGGGCATCATGTAG |
