# Supplementary material for: miR-422a inhibits cell proliferation in colorectal cancer by targeting AKT1 and MAPK1
Source: Cancer Cell Int. 2017 Oct 28;17:91. doi: 10.1186/s12935-017-0461-3 (PMC5664829; doi:10.1186/s12935-017-0461-3)
Supplement: Supplementary file 2 — Additional file 2: Table S2. Primer Sequences Used for Real-time PCR (5′ to 3′). [file 12935_2017_461_MOESM2_ESM.doc]

**Supplementary Table S2** Primer Sequences Used for Real-time PCR (5' to 3')

| **Gene** | **Forward primer** | **Reverse primer** |
| --- | --- | --- |
| GAPDH | GACTCATGACCACAGTCCATGC | AGAGGCAGGGATGATGTTCTG |
| CyclinD1 | GCTGCGAAGTGGAAACCATC | CCTCCTTCTGCACACATTTGAA |
| AKT1 | AGCGACGTGGCTATTGTGAAG | GCCATCATTCTTGAGGAGGAAGT |
| MAPK1 | ACCAACCTCTCGTACATCGG | GGGGCTGATTTTCTTGATAGC |
| GSK3β | CAACTGCCCGACTAACAC | GAGGAGGAATAAGGATGGTA |
| p27^Kip1^ | CCGGTGGACCACGAAGAGT | GCTCGCCTCTTCCATGTCTC |
| P21^Cip1^ | ACATCGCCAAGGAAAAACGC | GTCTGTTTCGGTACTGTCATCC |
|  |  |  |
