# Supplementary material for: miR-422a inhibits cell proliferation in colorectal cancer by targeting AKT1 and MAPK1
Source: Cancer Cell Int. 2017 Oct 28;17:91. doi: 10.1186/s12935-017-0461-3 (PMC5664829; doi:10.1186/s12935-017-0461-3)
Supplement: Supplementary file 3 — Additional file 3. Additional methods. [file 12935_2017_461_MOESM3_ESM.doc]

**SUPPLEMENTARY METHODS**

**Colony formation assays**

Cells (200cells/well) trypsinized were and plated on 6-well plates and cultured with medium (10%FBS) for 2 weeks. The colonies were fixed with 4% paraformaldehyde for 5 minutes and stained with 1% crystal violet for 30 seconds . We counted the colonies defined as >50 cells/colony. Three independent experiments were designed. The data was subjected to paired t test.

**Soft agar assays**

Cells (1×10^4^) were cultured in RPMI 1640 supplemented with 10% FBS with 0.3% agarose and layered on top of 0.6% agar in medium resuspended with 20% FBS on 60-mm plates. The plates were incubated in a humid atmosphere with 5% CO2 at 37℃. After 2 to 3 weeks, the number of cell colony were counted under microscope and cell colonies were photographed at a magnifica of 100. Only cell colonies considered >50 cells/colony were counted. The experiment was repeated for 3 times independently, each cell line respectively.

MTT assay

Cells were incubated for 24 hours at 37°C after the cells trypsinized and plated on 96-well plates (1×10^3^). Then 20ul of 5g/L MTT(3-(4,5-dimethylthiazol-z-yl)-2,5-diphenyltetrazolium bromide, Sigma, St Louis, MO, USA) was added and mixed into each well and incubated at 37°C.After 4h letter ,the MTT-medium mixture were removed and150 ul dimethyl sulphoxide (DMSO, sigma, St, Louis, MO, USA) were added into the wells. The absorbance value was measured at 490 nm with a Microplate Autoreader (Bio-Rad, Hercules, CA, USA). Three dependent experiment was repeated.
