# Supplementary material for: miR-422a inhibits cell proliferation in colorectal cancer by targeting AKT1 and MAPK1
Source: Cancer Cell Int. 2017 Oct 28;17:91. doi: 10.1186/s12935-017-0461-3 (PMC5664829; doi:10.1186/s12935-017-0461-3)
Supplement: Supplementary file 4 — Additional file 4: Table S3. Characteristics of 46 patients from dataset (GSE35834). Table S4. Sample set description. [file 12935_2017_461_MOESM4_ESM.doc]

**Supplementary Table S3. Characteristics of 46 patients from dataset (GSE35834)**

| **Characteristics** |  | **Number of samples** | **Percentage (%)** | |
| --- | --- | --- | --- | --- |
| Gender |  | |  |  |
| Female | 16 | | 35.6 | |
| Male | 29 | | 64.4 | |
| Total | 45 | | 100 | |
| Age(years,mean±SD) | 60.7 ± 10.2 | |  | |
| TNM stage |  | |  | |
| I | 2 | | 6.5 | |
| II | 1 | | 3.2 | |
| III | 1 | | 3.2 | |
| IV | 26 | | 83.9 | |
| Unclear | 1 | | 3.2 | |
| Table | 31 | | 100 | |
| Liver metastasis |  | |  | |
| Synchronous | 18 | | 75 | |
| Metachronous | 6 | | 25 | |
| Total | 24 | | 100 | |

**Supplementary Table S4. Sample set description.**

|  |  | **Number of samples** | **Percentage (%)** |
| --- | --- | --- | --- |
| Tissue type | N | 23 | 29.5 |
|  | T | 31 | 39.7 |
|  | M | 24 | 20.8 |
|  | total | 78 | 100 |
| Matched type | N-T | 7 | 15.6 |
|  | T-M | 8 | 17.8 |
|  | N-T-M | 8 | 17.8 |
|  | M-N | 2 | 4.4 |
|  | N | 6 | 13.3 |
|  | T | 8 | 17.8 |
|  | M | 6 | 13.3 |
|  | total | 45 | 100 |
