# Supplementary figures and images for: miR-422a inhibits cell proliferation in colorectal cancer by targeting AKT1 and MAPK1
Source: Cancer Cell Int. 2017 Oct 28;17:91. doi: 10.1186/s12935-017-0461-3 (PMC5664829; doi:10.1186/s12935-017-0461-3)

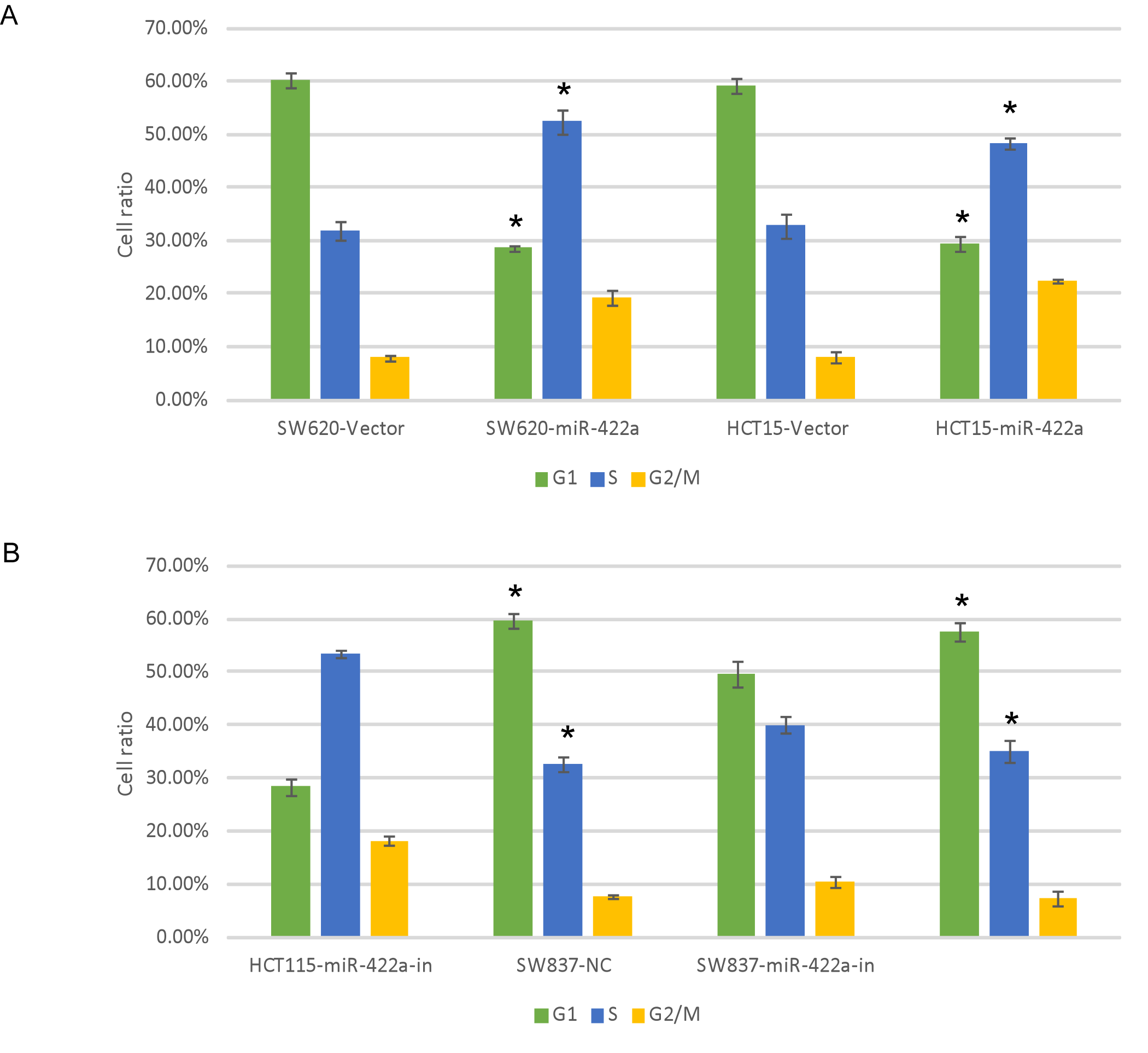

Supplement: Supplementary file 5 — Additional file 5: Figure S1. Statistical analyses of flow cytometry indicated that overexpression of miR-422a caused the arrest of G1-S phase transition (A), while knockdown of miR-422a promoted the G1-S phase transition (B). Error bars represent mean ± SD from 3 independent experiments. * p < 0.01. [file 12935_2017_461_MOESM5_ESM.png]
